# A Lightweight Hybrid Deep Learning Privacy Preserving Model for FC-Based Industrial Internet of Medical Things

**DOI:** 10.3390/s22062112

**Published:** 2022-03-09

**Authors:** Mohammed Amin Almaiah, Aitizaz Ali, Fahima Hajjej, Muhammad Fermi Pasha, Manal Abdullah Alohali

**Affiliations:** 1 Department of Computer Networks and Communications, College of Computer Sciences and Information Technology, King Faisal University, Al-Ahsa 31982, Saudi Arabia; 2 School of Information Technology, Monash University, Subang Jaya 47500, Malaysia; aitizaz.ali@monash.edu (A.A.); muhammad1.fermipasha@monash.edu (M.F.P.); 3 Department of Information Systems, College of Computer and Information Sciences, Princess Nourah bint Abdulrahman University, P.O. Box 84428, Riyadh 11671, Saudi Arabia; fshajjej@pnu.edu.sa (F.H.); manal@pnu.edu.sa (M.A.A.)

**Keywords:** security, IoT network, BLSTM, privacy, PoW, blockchain, smart contracts

## Abstract

The Industrial Internet of Things (IIoT) is gaining importance as most technologies and applications are integrated with the IIoT. Moreover, it consists of several tiny sensors to sense the environment and gather the information. These devices continuously monitor, collect, exchange, analyze, and transfer the captured data to nearby devices or servers using an open channel, i.e., internet. However, such centralized system based on IIoT provides more vulnerabilities to security and privacy in IIoT networks. In order to resolve these issues, we present a blockchain-based deep-learning framework that provides two levels of security and privacy. First a blockchain scheme is designed where each participating entities are registered, verified, and thereafter validated using smart contract based enhanced Proof of Work, to achieve the target of security and privacy. Second, a deep-learning scheme with a Variational AutoEncoder (VAE) technique for privacy and Bidirectional Long Short-Term Memory (BiLSTM) for intrusion detection is designed. The experimental results are based on the IoT-Botnet and ToN-IoT datasets that are publicly available. The proposed simulations results are compared with the benchmark models and it is validated that the proposed framework outperforms the existing system.

## 1. Introduction

**Figure 1:**
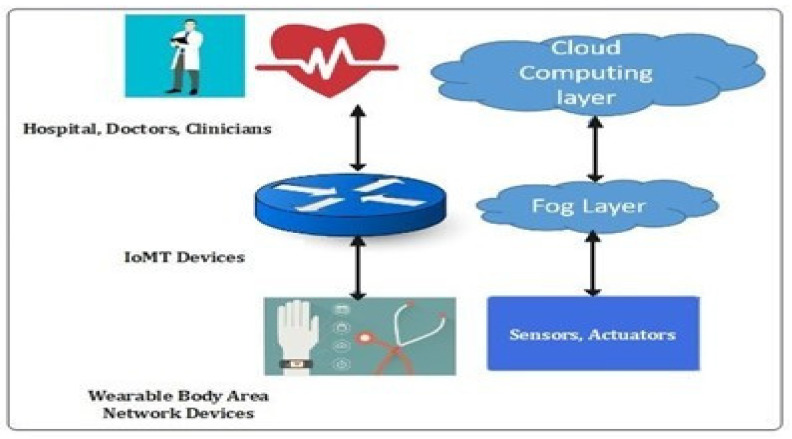
Application of fog computing.

The Internet of Things (IoT)-based applications and services include sensor networks, healthcare systems, transportation, smart industry, communication systems, smart cities, and manufacturing [1]. The Industrial Internet of Things (IIoT) has been proposed to dramatically enhance qualities of traditional industries, break regional limitations to achieve remote monitoring, perform autonomous production, and provide real-time information to users [2,3,4]. The Internet of Thing (IoT) will deliver about 85% of all IoT devices in healthcare by 2025 [1]. According to Tractia, an intelligent organization, annual earnings in this sector using blockchain technologies would reach USD 9 billion by 2025 [2]. IoT devices are widely used in healthcare to give real-time services to patients and physicians [3]. IoMT-based medical device applications include medical institutions and businesses. However, as the number of internet-connected medical devices (IoMT) increases, greater volumes and inconsistency of data will be generated. With centralized cloud-based characteristics, handling significant data traffic in IoT (IoMT) has now become a severe problem and reason for concern [4]. As a result, patient safety and confidentiality concerns have grown while data collection, data ownership, location privacy, etc., will be at risk. By copying data and changing the identification of healthcare equipment, intruders and hackers can easily target the 5G-enabled IoMT network. IoMT-Cloud currently has a single point of failure, malicious attacks, and privacy leaks, as shown in Figure 1. To ensure network security and secure PHR transmission, data transfer between IoMT and Cloud requires trust, device identification, and user authentication (UA). With the traditional Central Cloud service, however, due to the round-the-clock networking of nodes in this IoT network, it is vulnerable to various security issues, such as message tampering, eavesdropping, and denial-of-service attacks [5]. In the industrial industry, this raises major security issues as the misuse of data can result in the incorrect diagnosis and can cause life-threatening scenarios for the patients under observation [6,7].

## 2. Background and Related Studies

The fog computing-based IoMT is currently a popular topic. Previous research missed important security issues such as: 1. Healthcare IoMT devices send data to cloud servers that are frequently unencrypted and open to manipulation and attack. As a result, sensitive patient information will likely be accessible. This issue leads to security vulnerabilities. 2. To our knowledge, the need to identify IoMT medical devices, which leads to the verification and authentication of health data, is considered very important and sensitive, and it can be accomplished quickly using a blockchain in the FC-IoMT system. Moreover, servers at the network’s edge should perform more detailed authentication and verification. BAKMP-IoMT, the new IoMT key agreement technique for blockchain-accessible authentication, was designed by [8]. It is also obtained theoretically from the algorithm’s top time complexity and the number of patients. Researcher in a study [9] explored various design research topics on readers’ 5G-enabled tactile internet fog computing. In the same way [10] thoroughly examined 5G-assisted smart health (Version 30 November 2021; Journal submitted to: not specified) of 14 care solutions in the IoT. R. Researchers in a study [11] proposed a multi-cloud cascade architecture, a low-overhead native testing framework, and a medical data storage backup method. This is also something that is examined by researchers [12] proposed a smart authentication (SSA) system to improve patient–physician data security and privacy preservation systems. Ref. [13] designed a node security identity authentication; providing a secure and reliable updating method for authentication keys and session keys. Ref. [14] proposed smart remote healthcare systems that require long working periods, low cost, network resilience, and confidence in highly dynamic network environments. Ref. [15] highlight the rising issues in IIoT information processing storage, querying, and dynamic data collecting. Researchers in a study [16] proposed a 69 case database and the current patient’s privacy was protected regardless of whether the abstracts matched or not. The overall comparative analysis of different parameters for 5G-enabled IoMT communication such as IoMT application, scalability, security, sustainability, storage, and computing is presented in Table 1. Ethereum is a permissionless blockchain that has been widely used by various blockchain enthusiasts. Ethereum follows the standard principles and elements of a blockchain network. Similar to a84blockchain, it uses the Proof of Work (PoW) consensus process to facilitate validation of blocks of the network by mining nodes before adding the blocks and their transactions into the blockchain. Ethereum can be utilized by connecting nodes to a blockchain with a unique chain-id. This allows all the nodes to participate within the blockchain activities and access blocks and/or transactions. Ethereum can also be implemented as a private blockchain for typical enterprise solutions that restrict access to their trusted assets and personnel [17].

A recent study conducted by Dorri et al. [18] reviewed the main challenges of fog computing and IOT. The study concluded the recent trends of IOT algorithms and the main challenges in fog computing, which works as a middle layer between data centers in the cloud and IOT networks. Hang et al. [19] developed a new scheme that captures the most significant features of the DBMS environment, including relational, graph-based, key-value, tree-like, etc., query languages, platforms (servers), plus running environments (desktop, Web, cloud), and specific contexts—i.e., focusing on optimizing queries, redundancy, security, performance, as compared with other schema-less approaches, programming languages/paradigms, and others. Yu et al. [20] focused on Quality of Service (QoS) in IOT utilization. They performed an analysis review on QoS techniques developed in the literature for IoT applications and investigated current research trends. They found that the most popular QoS metrics are Network Usage, Throughput, Reliability, and Latency.

### 2.1. Smart Contracts

The classical distributed consensus mechanism is the consensus mechanism used in the traditional distributed network, which realizes the distributed consensus through the state machine replication between network nodes. Hameed et al. [21] proposed the Byzantine Generals Problem and studied how non-fault nodes reach agreement on specific data in the case of possible failure nodes or malicious attacks, which became the basis for the research on consensus mechanisms. Dwivedi et al. [22] proposed a Paxos algorithm to solve the Byzantine Generals Problem. This algorithm can tolerate the collapse of a certain number of nodes in the network, so as to reach an agreement on a specific value in the distributed system. Daraghmi et al. [23] proposed the Practical Byzantine Fault Tolerance (PBFT). As a solution to the Byzantine Generals Problem, PBFT could achieve the final consensus among honest nodes while the number of enemies was no more than 1/3 of the total number of nodes. Jung et al. [24] proposed a new common algorithm: Mixed Byzantine Fault Tolerance (MBFT). Functionally, MBFT partitions the nodes participating in the consensus process and improves scalability and efficiency without sacrificing security. MBFT also introduces a random node selection mechanism and a credit mechanism to improve security and fault tolerance. Esposito et al. [25] proposed a dynamic reputation practical Byzantine fault tolerance algorithm. The dynamic reputation practical Byzantine fault tolerant algorithm adopts the consensus election method based on credit. The monitoring node divides the remaining nodes into two types of nodes according to their reputation values: consensus nodes and auxiliary nodes, which participate in different stages of the block generation process, respectively, and dynamically update the consensus nodes with low reputation scores.

### 2.2. PoX Consensus Mechanism

The PoX consensus mechanism is usually a blockchain consensus mechanism oriented towards the public chain. Its core idea is to determine the probability and expectation of the nodes to obtain the correct accounting based on the proportion of certain key resources owned by the nodes, so as to improve the security of the public chain network. Kermanshahi et al. [26] realized the design of a bitcoin system based on the traditional Proof of Work (PoW), and the blockchain was proposed for the first time as its underlying technology. Kermanshahi et al. [27] proposed Proof of Stake and introduced the concept of age of currency for the first time. The core idea is that the more coins a node has and the longer it has been holding coins, the more likely it will be chosen as a blocker. Kermanshahi et al. [28] proposed Permacoin based on Proof of Capacity (PoC), which requires participants to be able to store part of a large file. The authors of [18] proposed a novel lightweight Proof of Block Trade (PoBT) algorithm for the blockchain of the Internet of Things and its integrated framework, which can verify transactions and blocks with reduced computing time. Kermanshahi et al. [29] proposed a novel consensus mechanism called Proof of Negotiation (PoN). PoN introduced a trust mechanism to realize the random selection of honest miners and conducted a round of block creation through a negotiation mechanism.

### 2.3. Authorization Consensus Mechanism

The main idea of the authorization consensus mechanism is to complete the generation and maintenance of blocks through a distributed consistency algorithm after nodes have been authenticated. Dwivedi et al. [30] proposed the basic framework for Hyperledger Fabric. Hyperledger is a series of open source blockchain projects initiated by the Linux Foundation, which aims to provide an enterprise-class open-source distributed ledger framework and source code. Hyperledger Fabric is a community-based project that provides a supporting framework for blockchain applications. Rathi et al. [31] proposed the DFINITY consensus mechanism. DFINITY protocol operates in periods and divides all participating nodes into different groups. A random committee is responsible for transaction processing and consensus operation in each period, and at the end of each period, a random number function is used to determine the group serving as the committee in the next period. The PaLa consensus mechanism proposed by [32,33,34,35,36] realizes the rapid consensus in the authorization network. PaLa uses the method of parallel pipeline to improve the efficiency of block processing and adopts the sub-committee sliding window reconfiguration to ensure the sustainability of transaction processing during the reconfiguration.

### 2.4. Hybrid Consensus Mechanism

The main idea of the hybrid consensus mechanism is to select some nodes as the consensus committee through the PoX consensus mechanism and run the Byzantine consensus mechanism inside the committee to complete the generation of blocks. Ali et al. [37] first combined the classical distributed consistency algorithm PBFT with blockchain and proposed the PeerCensus consensus algorithm. Bitcoin is used as the underlying chain to select a certain number of nodes and complete the generation of the final block through the Chain Agreement (CA) algorithm after their identity authentication. Ali et al. [38] proposed the Hybrid Consensus mechanism, which realized state machine replication in an unauthorized environment by using workload proof. Hybrid Consensus for the first time uses a formal security model and modular design to model the hybrid consensus mechanism and proves that it can meet the safety characteristics such as consistency and activity. Siam et al. [39] proposed ELASTICO, a fragmentation consensus mechanism, which divides nodes participating in the consensus into multiple groups, outputs a block from each group, and then obtains the total block. Qasem et al. [40] proposed the RapidChain consensus mechanism, which realized computing sharing, communication sharing, and storage sharing. Its main modules include startup, consensus, and reconfiguration. Almaiah [41] proposed a Proof of QoS (PoQ) based on Quality of Service (QoS). In this validation protocol, the whole network is divided into several small regions, each region specifies a node according to its QoS, and then runs deterministic Byzantine fault tolerant consensus among all the specified nodes. Although the above-mentioned consensus mechanisms on the indices such as security and efficiency have excellent performance, but the consensus mechanism is still facing single-chain or homogeneous blockchain, they cannot be directly applied to multilevel heterogeneous and cross-blockchain application scenarios of governing blockchain by blockchain. They still need a safe, efficient, and scalable cross-blockchain mechanism for governing blockchain by blockchain frameworks. One of the key distinguishing features that hyper ledger supports for its users is smart contracts. The concept of smart contracts was introduced by Nick Szabo in 1994 who defined it as “a computerized transaction protocol that executes the terms of a contract”. The primary objective of introducing smart contracts was to facilitate the execution of scripts stored in the blockchain without the need for an intermediate entity. 

Transactions: a transaction is a signed package of data that contains the following components: the signature of the sender and recipient of the message. The amount of ether to be transferred to the data field (optional). GASPRICE: fee required per computational step required for the sender to pay. STARTGAS: represents the max number of computational steps allowed for the transaction to execute. The data field is the key field that the contracts use to read whenever a smart contract is addressed [42,43,44,45,46]. IIoT Security Threats IIoT solutions consist of industrial systems that connect to cloud for data collection and analysis purposes. IIoT is similar to the traditional Industrial Control Systems (ICS) although the tight security restriction applied in ICS cannot be applied to IIoT environments naturally. This is due to the necessity of cloud computing requiring IIoT devices to have direct access to the internet. This is different from the traditional ICS environments that require different zoning and in-depth defense frameworks. ICS environments differ from the standard enterprise environments in many ways, and below are some of the key differences [47,48,49,50]. Risk level: significant impact on human lives and possibly the nation. Performance Requirements: requires real-time analysis as performance issues can affect the operations and hence can be risky to the organization. Availability Requirements: needs to be available at all times with redundant systems to ensure availability in case of a failure. Safety: safety requirements to be able prevent hazards by detecting unusual behavior and triggering alarms and safety measures. Multiple attacks have occurred in the past using different attack vectors using malware payload to take control of the ICS system. This includes but is not limited to the following ICS cyber attacks: 1. Stuxnet Malware [50,51,52,53,54,55]. Multiple attacks on the Ukraine Power Grid in 2015 and 2016 [56,57]. 2. Ransomware attacks by NotPetya [18]. TRITON attack framework targeting the safety instrumented system [58,59,60]. By analyzing the requirements and the risk level of traditional ICS environments, we can see that the data these systems transfer and receive within these environments are considered to be very sensitive. Any data exposure to unauthorized parties can have a major impact on organizations and potentially nations as a whole. Implementing IIoT may involve exposing some of these sensitive systems directly to the cloud. There have been multiple incidents that involve compromising IoT devices in order to use them to launch DDoS attacks [61,62,63] or to breach data to Command-Control (C2) servers [64,65,66,67]. By performing appropriate threat modeling of a 150private Ethereum blockchain solution, our paper examined the following research questions:

1. What are the threats that IIoT will face when blockchains are utilized in their environments?
2. How can blockchain transparency impact the exposure of IIoT environments to external threats?
3. What are the implications of compromising blockchain nodes within IIoT environments?

### 2.5. Contribution

The following are the main contributions of this research. 1. A novel proposed a scalable blockchain architecture for FC-based 5G-enabled IoMT that considers secure data access (SDA) and trust. 2. The integration of fog-based IoMT with a unique and decentralized management confidence architecture based on blockchain technology. 3. The use of a lightweight encryption system to reduce the computational, storage, and communication overhead. The rest of the paper is organized in the following structure: Section 3 describes the related work undertaken by the researcher. Section 3 has two subsections: (A) that overviews the 5G-enabled IoMT; (B) blockchain and fog-based architecture for IoMT. Section 4 represents the simulation and analysis of results. The last Section 5 and Section 6, represents the conclusion and future work.

## 3. Methodology

**Figure 2:**
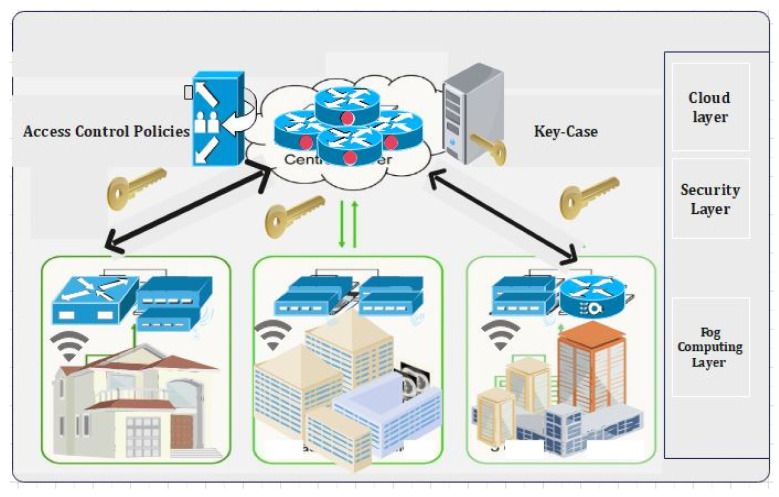
System layers of the proposed model.

The proposed research methodology consists of training the proposed hybrid deep-learning model in a distributed manner and then deploying it on the edge devices. The edge or fog devices use its local data to update the pre-trained model and evaluate their own models. Moreover, all the users in the proposed model are considered trusted users due to the application of blockchain technology [1,6,7]. Due to the secured and flexible access control scheme, maintaining data integrity when communicating the data over IoT network is a challenging issue. Second, designing an adaptable security mechanism that can efficiently distinguish normal and attack instances in IIoT is also a challenging issue. The proposed model consists of an IoMT network consisting of various interconnected medical sensors, actuators, and machines, located at multiple fog nodes [8,10,14]. Third, developing a new framework for deploying blockchain and deep-learning techniques in current cloud-edge assisted industrial systems is of utmost importance with the integration of blockchain. The backup data is stored in the cloud whereas the meta-data are stored and hashed inside the blockchain. As such framework often faces issues related to scalability, due to different computing power of the participating edge nodes it is infeasible to store the complete block in the edge networks [4,5]. Figure 2 represents the scenarios of our proposed framework. The integration of blockchain with the hybrid IOT and its application in fog computing are explained in Figure 2.

**Figure 3:**
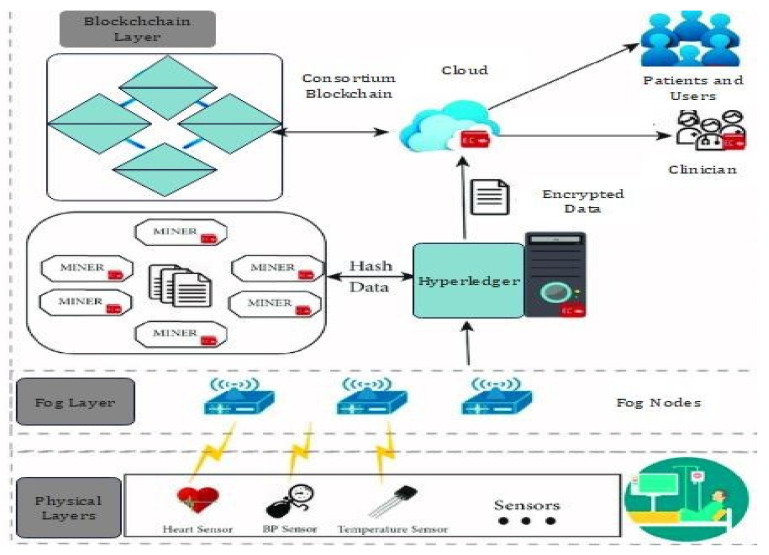
Integration of the blockchain with the proposed model.

This is based on the blockchain concept and can be utilized to perform peer-to-peer data transmission in a secured manner. This will be used to store transaction data for a long time and will not be fabricated or deleted from the blockchain. Furthermore, the transaction details are kept on the cloud server, which makes the data immutable and decentralized [12]. The data integrity can be achieved by the secure hash function SHA512. This hash function is included in the respective message digest and creates the fixed length of the unique fingerprint. The used SHA512 is resistant to collision and can be applied in real-time processing along with brute force attacks [13]. The message digest can be used in transaction blocks since it can circumvent the poisoning attack known as the avalanche effect [14]. Moreover, if we change one bit of data this can completely change the message digest. Thus, it preserves the IoT data integrity. Some of the information present in the created blocks are block index, previous hash, current block hash, current proof, data (Tvalue), (Tscore), and timestamp. Figure 3 represents different modules of the proposed system architecture and the flow of data through various organizations.

**Figure 4:**
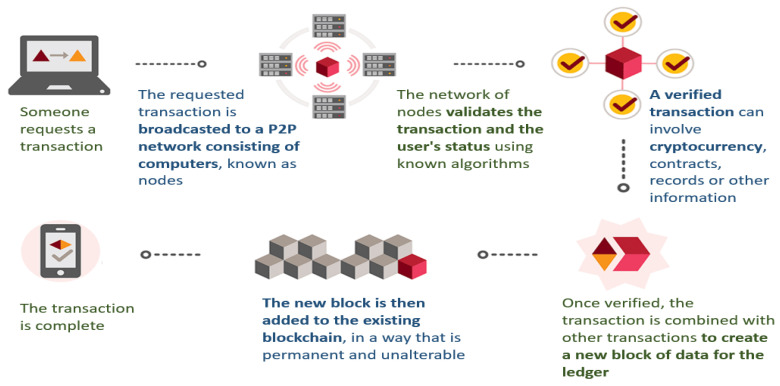
Flow of data through the proposed model.

**Figure 5:**
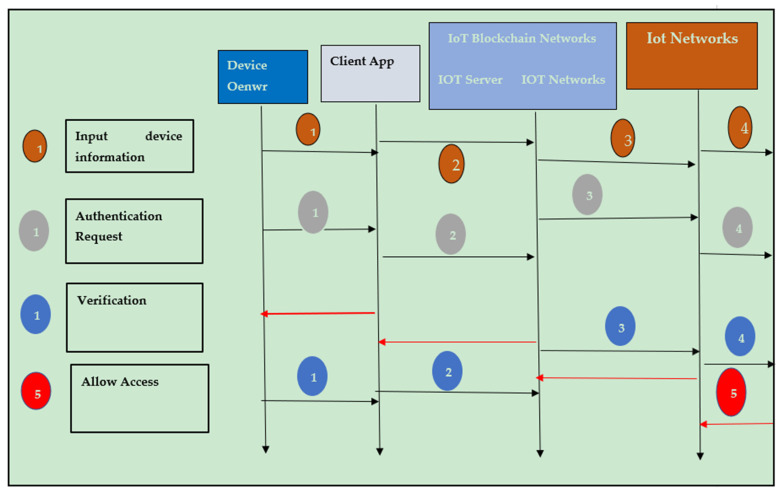
Timeline diagram of the proposed model.

As shown in Figure 4 and Figure 5, the generated blockchain is maintained with the help of the hash function of the previous chain. Thus, the verifiability of the system has been enhanced. Moreover, in the blockchain, the verification of the integrity of the hash chain has been performed while conducting the generation of new blocks by utilizing the consensus mechanism [15]. 5G-Enabled IoMT Communication. One of the essential applications in 5G networks is smart healthcare. The general architecture and essential entities of the 5G intelligent health network are depicted in Figure 2, representing the smart antenna requirement for 5G-enabled network communication. Smart antennas benefit from several significant advancements in the current scenario that boost 5G [8]. Perfect signal and transmission capabilities are possible thanks to a well-coordinated RF beam. However, because the focus of interest diminishes with increased attenuation, the location remains an issue. The use of machine-to-machine connections (M2M) and the IoMT as the foundations of intelligent healthcare in 5G networks (IoMT) is predictable. There are two fundamental drawbacks to the strategies given. The first is many terminals, resulting in dense networks. For IoMT and M2M applications, ultra-density and scalability issues are required. The second point of concern is secure consumption, which results from the nature of IoMT-based [9] applications that use wireless sensors. Figure 5 describes the timeline diagram of the proposed model and its function.

## 4. Proposed Framework

This section presents the proposed framework for fog computing using 5G technology. Once the data is generated via IoT devices, the network traffic is routed to FogBlock by the nearest gateway/router. The incoming traffic is sniffed using a sensor, which extracts features at the fog block. The reputation score is computed, and the address-based blockchain reputation system is designed. The three primary classifications of transactions based on the outcome are general, honest, and dishonest. The distributed file storage system stores the raw or transaction data [25]. The raw data was transmitted with trust information using the privacy-preservation module and the hast proof with message digest is constructed using ePoW on the blockchain which prevents inference attacks and validates data record chains using system-based machine learning. The GTBSS-HDNN model categorizes many types of attacks as well as normal data. The MICA approach uses second-level privacy to transform the original data into a new transformed format. The anomalous class administrator is replaced at the end and the Cloud Block receives the request safely.

In Cloud Block, several providers offer various types of data centers. In the proposed architecture, three data centers are used: A, B, and C. To create a blockchain network, the 268 proposed GTBSS-HDNN architecture is implemented at each data center and these are the 269 entities in the Cloud Block network [26]. The trust between the verifiable, auditable, 270, and immutable blocks is built using the proposed hybrid NN architecture. Threat ID type properties: T1 Internal Confidentiality; T2 External Availability Internal Threats. The likelihood of internal threats mainly depends on the frequency of security attacks and data breaches caused by insider threats. According to a report produced by Nucleus Cyber in 2019 [31], 60% of the surveyed organizations had experienced one or more insider attacks within the last 12 months in 2019. In addition, according to a threat report produced by Proof point [32], around 75% percent of the reported attacks analyzed were caused by criminal or malicious insiders in 2020. Therefore, the threat the likelihood for threat T1 can be considered to be moderate. External threats: A shown in Figure 4, the intended architecture is composed of several layers. By processing IoMT data on fog nodes (FN), the initial layer (IL) of FN minimizes latency. This also enables the user to realize his desire for quick service. In future, in IoMT devices [25], a multi-layered design, as depicted in Figure 2, has been proposed for applications involving large amounts of data. The devices connected and FN are shown in the first layer of this design. Connected devices communicate with one another, and blockchain technology provides security. The second level of FN’s latency is reduced because of IoMT device communication. As a result, users’ requirements are encountered in the proposed fog computing (FC) model [43,44,45,46,47,48,49,50].

### 4.1. Proposed Smart Contracts and Fog Computing

**Figure 6:**
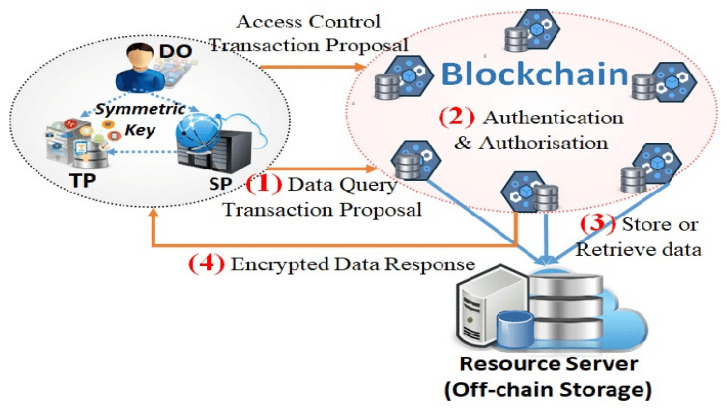
Proposed access control and the data flow through blockchain.

**Figure 7:**
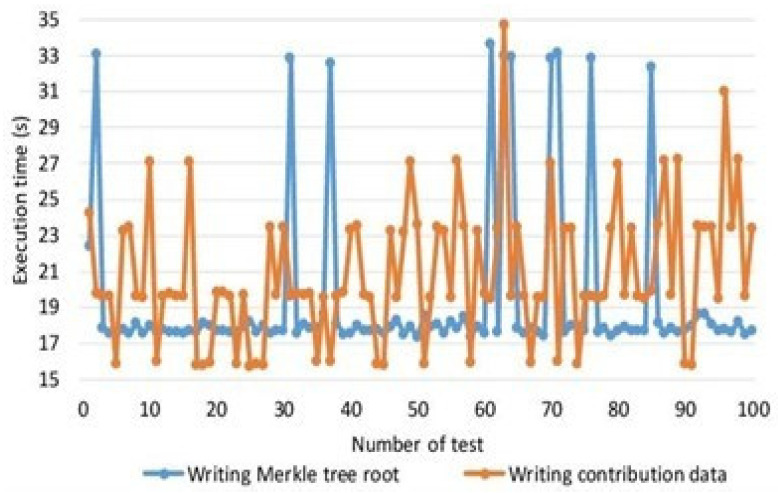
Comparative analysis of the writing Merkle tree vs. writing contribution data.

**Figure 8:**
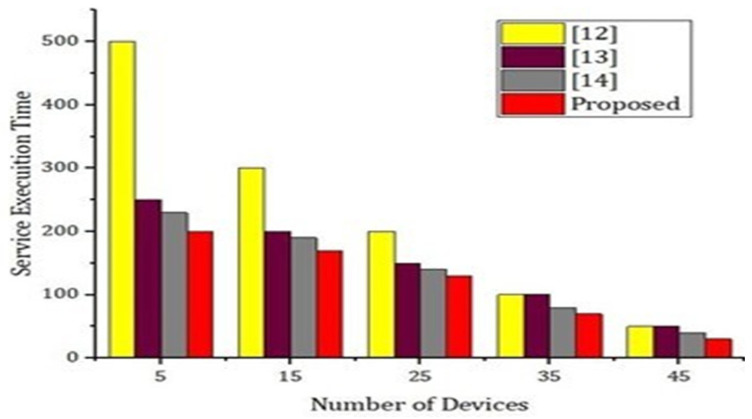
Performance evaluation of the proposed model versus the benchmark model.

A blockchain and fog network [24] connects the Internet of Medical Things (IoMT) and fog nodes (FN) (IoMT-Fog). Distributed technology can deliver on-demand services by combining high performance and low latency (LL). It will raise the threshold for monitoring people’s health. The FC paradigm aids IoMT elements with low latency (LL), allowing for faster data processing. The proposed IoMT-Fog, shown in Figure 6 and Figure 7, could provide a more appropriate medical equipment (ME) solution. The proposed neural network with the integration of smart contracts is represented through Figure 8.

One of the essential applications in 5G networks is smart healthcare. The general architecture and essential entities of the 5G intelligent health network are depicted in Figure 2, which represents the smart antenna requirement for 5G-enabled network communication. Smart antennas benefit from several significant advancements in the current scenario boost 5G coverage and capacity. Beam shaping (vertical and horizontal) is a breakthrough that concentrates RF energy in a compact beam and targets it precisely where it is needed, rather than dispersing it over a large region. 

### 4.2. Proposed Algorithm

In this section we describe our proposed two algorithms (Algorithms 1 and 2). These algorithms are represented as below:
**Algorithm 1: FC-Average Algorithm***1: Init: a = 0;* *2: for each round t = 1,2,…do* *3: select K clients* *4: for each K clients do* *5: w^k^t, UpdateClient( )* *6: d^k^t* ← *the distance between two classes dataset end for* *7: If (d^k^ = t)*  ←*1* 8: d^k^ = w^k^ * t   ←*p^k^ k = 1nk f(d^k^t) wt1/pkk = 1nkf^d^ kt)* *9: end for* *10: Updatefunction( )* *11: Initialize local minibatch size L, local epochs E, learning rate* *12: for each epoch i E do* *13: randomly choose S: based on size L* *14: wi* ←   *w1 – w5g(w1:s)* *15: end for* *16: return i* *17: End Procedure* *18: End Algorithm*



**Algorithm 2: Algorithm Method Evaluation**
*1: Enhance Analysis of both the IOMT end* *2: Select IOMT node for Transaction selection (Node)* *3: Get EMR data, hash, get (EMR)* *4: Extract EMRFromRepository from ERM (ERM name)* *5: ERM, valid SHA256 CheckHash (ERM, Hash)* *6: if ERM is T, then* *7: Get the Connent (Connect)* *8: Generate Indications (Connect length)* *9: Valid Blockchain transaction Valid (i, indications)* *10: Del Local EMR delete (EMR)* *11: End if (EMR)* *12: End* *13: End* *14: wi* ←  *w1 – w5g(w1:s)*

## 5. Experimental Setup

We set up an experimental environment to implement our proposed framework using Fog Node (FN) for IoMT networks with the corresponding throughput elapsed time or intervened time. When a cloud-based ordering system is established using a virtual machine (VM), the time of the associated bypass and the number of nodes is assessed. For performance measurement, 16.04 LTS Core i5 CPU Ubuntu is a Linux distribution 2.50 GHz 2.71 GHz (VirtualBox). The RAM capacity is 16 GB. Each follower peer virtualization scenario has 30 vCPUs and 8 gigabytes of RAM. It repeats the following process 30 times. The number of transactions handled per subsequent is referred to as a second transaction—several transactions per second (TPS). The response time was 226 milliseconds, with a minimum of milliseconds and milliseconds. Figure 6 shows the hyperledger-based fog architecture for intervened time. As the thread group starts and pauses a demo application, we notice network latency (NL). We also managed threads in a blockchain (BC) network successfully. The response times to the blockchain (BC) network are depicted in Figure 7 and show the intervened time and active thread in the fog computing environment. We moved the ordering service to the cloud to determine if the network is steady. Despite the low minimum (LM), the ordering instance 233 generates a reasonably stable network. It was proven to work in situations where 234 high throughput and a real-time environment are required. The performance graph of a 235 fog network based on Hyperleader Fabric (HF) over time is shown in Figure 7. Blockchain, IoMT (Fog-BC-IoMT), and FC technologies all utilized the proposed architecture (Fog-IoMT). To record transactions, the BC is utilized to create a legal public, hyperdistributed EMR. Several IoMT-NODES are utilized in the architecture testing and implementation. The outcomes were estimated satisfactorily. This study suggests an architecture for preventing data fraud by converting existing centralized database systems to block-based distributed databases. It divides the system into four parts: cloud, fog, blockchain, and IoMT. The IoMT system is self-contained. We also examined whether the network convention method could assist with public cloud resources more effectively. The ordered migrates to the cloud for stability, security, and scalability and avoids performance issues by not directly connecting IoMT devices to forbidden networks. Hyperledger, a chain block solution, handles IoMT validation and safety. To reduce network latency (NL) and output, smart contracts (SC) and transaction checking on fog nodes (FN) is recommended. The network architecture will challenge cost reductions in the cloud and optimize cloud and FN instancing performance to boost the efficiency of the hyperledger BC network [50,51,52,53,54,55,56,57,58].

## 6. Results and Discussion

**Figure 9:**
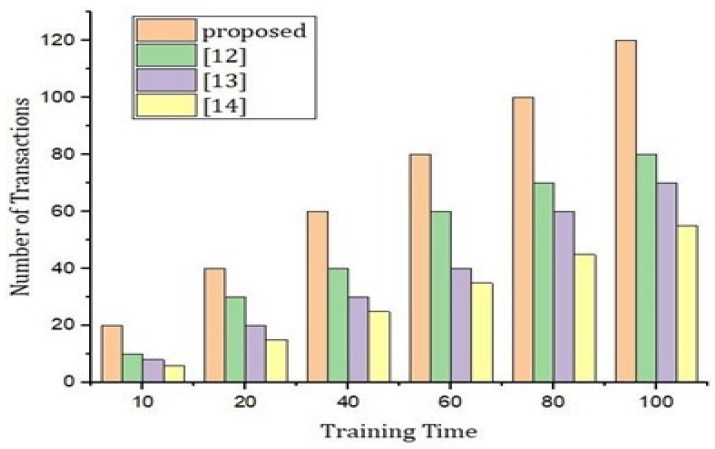
Performance analysis of the proposed system versus benchmark model.

**Figure 10:**
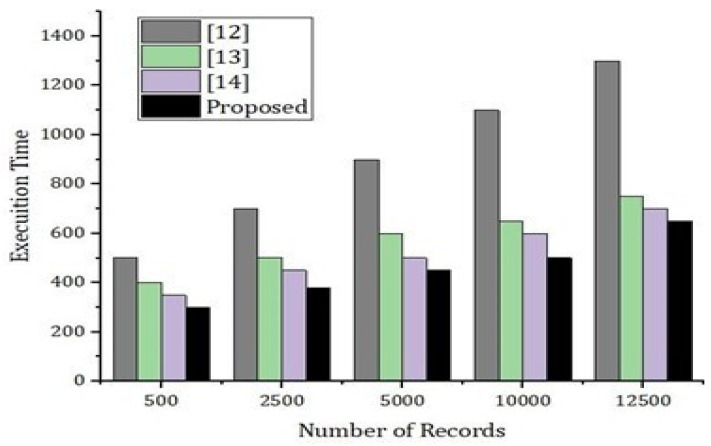
Latency evaluation through number of rounds versus execution time.

Internal threats: the likelihood of internal threats mainly depends on the frequency of security attacks and data breaches caused by insider threats [59,60,61,62,63,64,65,66,67]. According to a report produced by Nucleus Cyber in 2019 [31], 60% of the surveyed organizations have experienced one or more insider attacks within the last 12 months in 2019. In addition, according to a threat report produced by Proof point [32], around 50% percent of the reported attacks analyzed were caused by criminal or malicious insiders in 2020. Therefore, the likelihood for threat T1 can be considered to be moderate. Algorithm 2 describes the process of encryption and hashing techniques. We carried out the experimental setup based on the proposed framework and algorithm. The simulations were carried out using the hyperledger fabric tool. In order to implement the proposed algorithm and smart contracts we used chain code for blockchain transactions. The parameters used for analysis include block creation, encryption time, decryption time, number of transactions, number of iterations, and number of nodes. Figure 9 shows the comparative analysis between the writing Merkle tree root and writing contribution data using hybrid deep-learning techniques using consortium blockchain in terms of number of tests carried out and execution time in seconds. We carried out up to 100 tests and the execution time was noted as up to 100. Figure 10 simulations were carried out on the experimental results from the number of devices and the service execution time. We provided a comparative analysis through the experiment based on a simulation of the proposed model and the benchmark model. From Figure 7, it is very obvious that our proposed model takes significantly less process and execution time as compared to the benchmark models.

As presented in Figure 8, we carried out the simulations results based on training time based on the proposed hybrid deep-learning protocol (BLSTM + CNN) and the number of transactions. From the simulations results, it is very clear that the number of transactions is higher as compared to the benchmark models based on the training time. The simulation result in Figure 8 explains that our proposed system is more intelligent, and it transfers the blocks according to the requirements and quick access to the participants in the system, thus, it is time effective as well. 

**Figure 11:**
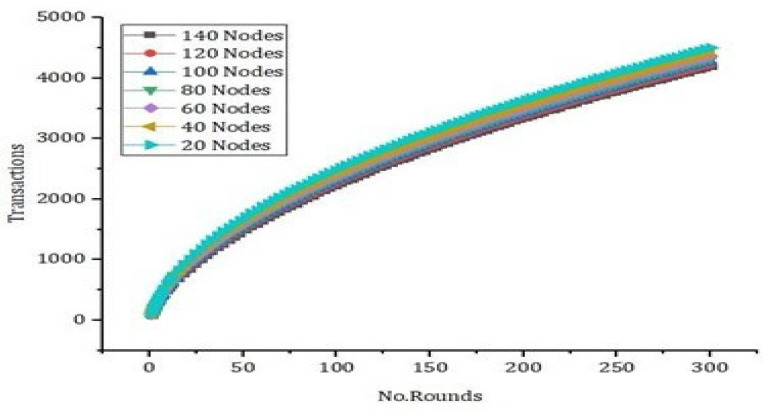
Comparative analysis of the number of rounds versus execution time.

As presented in Figure 9, we carried out simulation results based on number of records and the execution time. In Figure 11, we compare our proposed model with the benchmark model such as [12,13,14]. Using hybrid deep-learning techniques and choosing the blocks according to the requirements, our proposed model takes significantly less time as compared to the benchmark model. Thus, Figure 9 justifies that our proposed framework is efficient and intelligent. In Figure 10, we present an experimental analysis based on the number of rounds and the number of transactions through the proposed model. We tested our proposed model based on different nodes and we started from 20 nodes up to a maximum of 140 nodes. The number of rounds taken into account were 300 and the number of transactions counted was up to 5000.

**Figure 12:**
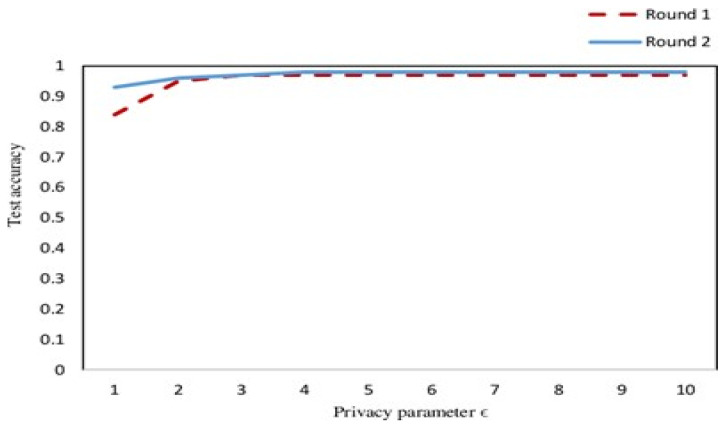
Privacy evaluation of the proposed model.

**Figure 13:**
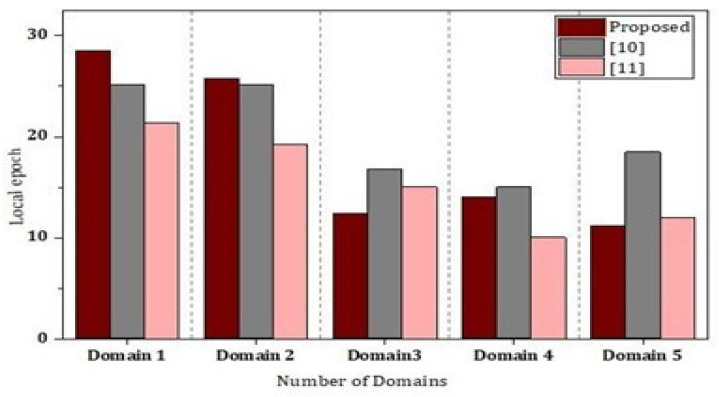
Cross-domain analysis of the proposed framework.

**Figure 14:**
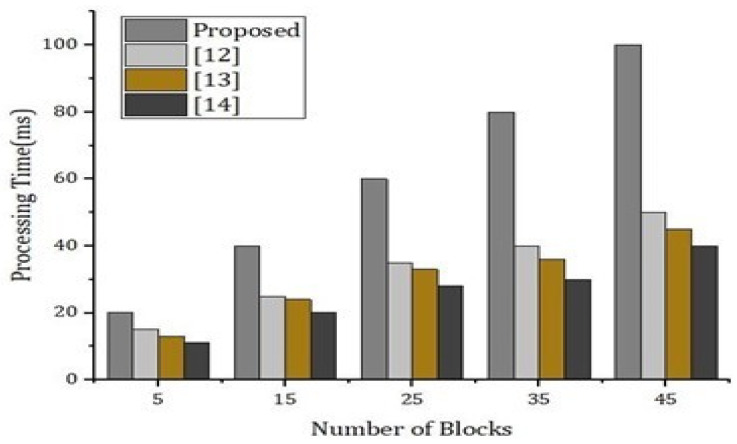
Effective cost analysis, w.r.t., number of blocks.

As presented in Figure 11, we carried out simulations based on two parameters including privacy parameter and test accuracy. We carried out the simulation results for two rounds and then we analyzed the test accuracy. We validated our simulations results through Figure 11, and we found that there is significantly less difference in the accuracy, showing the validation of our method for security and privacy. Figure 12 and Figure 13 represent the simulations results based on the number of domains and the local epoch. The value epoch represents the training of the datasets in batch form. Figure 14 represents the simulation results based on number of blocks and the processing time in microseconds.

## 7. Conclusions

In this research, a novel approach based on hybrid deep learning (BLST + CNN) was used to train the model in a decentralized fashion with minimum latency as well as less computational cost. The proposed model learns from a defined model which tracks the behavior and integration of the users. The proposed framework provides a decentralized nature and privacy preservation approach. The training of the model was carried out on each local device using hybrid deep learning (BLSTM + CNN). The bi-linear long short-term memory (BLSTM) consists of two modules, i.e., feed forward and feed backward and at the end it concatenates. We used the datasets based on IoT-ToN available publicly on UNSW, Australia website. Moreover, we divided the dataset into two parts, i.e., training and testing. Similarly, for the proposed model, 30% of data were used for training and 70% used for testing and validation. From the simulations results, it was concluded that the proposed model outperformed the benchmark model. The latency of the proposed framework was observed up to 20 ms which is lower as compared to the benchmark models. In order to provide privacy preservation, the proposed model was encrypted using lightweight encryption and decryption based on homomorphic encryption. Similarly, the use of homomorphic encryption provides the ability to perform additive or multiplicative operations over encrypted data. The proposed model is recommended for cross-domain networks in any healthcare systems. In the future, we want to extend the proposed research work using a PSO algorithm integrated with federated learning. This approach will improve the existing work.

## Figures and Tables

**Table 1:** Experimental Setup.

Component Name	Description	Types
Hardware	Raspberry Pi	Hard
Memory	1 GB	RAM
OS	Android	V.8
Language Tool	Java	Hyperledger
Simulation Tool	Mat lab	V.2020
Design Tool		Rational Rose
Editing Tool	Latex	V3

## Data Availability

The data used within the research can be provided by the first author upon request.
